# Mechanisms of Vascular Damage by Hemorrhagic Snake Venom Metalloproteinases: Tissue Distribution and *In Situ* Hydrolysis

**DOI:** 10.1371/journal.pntd.0000727

**Published:** 2010-06-29

**Authors:** Cristiani Baldo, Colin Jamora, Norma Yamanouye, Telma M. Zorn, Ana M. Moura-da-Silva

**Affiliations:** 1 Laboratório de Imunopatologia, Instituto Butantan, São Paulo, São Paulo, Brasil; 2 Section of Cell and Developmental Biology, Division of Biological Sciences, University of California San Diego, La Jolla, California, United States of America; 3 Laboratório de Farmacologia, Instituto Butantan, São Paulo, São Paulo, Brasil; 4 Laboratório da Biologia da Reprodução e Matriz Extracelular, Instituto de Ciências Biomédicas, USP, São Paulo, São Paulo, Brasil; Liverpool School of Tropical Medicine, United Kingdom

## Abstract

**Background:**

Envenoming by viper snakes constitutes an important public health problem in Brazil and other developing countries. Local hemorrhage is an important symptom of these accidents and is correlated with the action of snake venom metalloproteinases (SVMPs). The degradation of vascular basement membrane has been proposed as a key event for the capillary vessel disruption. However, SVMPs that present similar catalytic activity towards extracellular matrix proteins differ in their hemorrhagic activity, suggesting that other mechanisms might be contributing to the accumulation of SVMPs at the snakebite area allowing capillary disruption.

**Methodology/Principal Findings:**

In this work, we compared the tissue distribution and degradation of extracellular matrix proteins induced by jararhagin (highly hemorrhagic SVMP) and BnP1 (weakly hemorrhagic SVMP) using the mouse skin as experimental model. Jararhagin induced strong hemorrhage accompanied by hydrolysis of collagen fibers in the hypodermis and a marked degradation of type IV collagen at the vascular basement membrane. In contrast, BnP1 induced only a mild hemorrhage and did not disrupt collagen fibers or type IV collagen. Injection of Alexa488-labeled jararhagin revealed fluorescent staining around capillary vessels and co-localization with basement membrane type IV collagen. The same distribution pattern was detected with jararhagin-C (disintegrin-like/cysteine-rich domains of jararhagin). In opposition, BnP1 did not accumulate in the tissues.

**Conclusions/Significance:**

These results show a particular tissue distribution of hemorrhagic toxins accumulating at the basement membrane. This probably occurs through binding to collagens, which are drastically hydrolyzed at the sites of hemorrhagic lesions. Toxin accumulation near blood vessels explains enhanced catalysis of basement membrane components, resulting in the strong hemorrhagic activity of SVMPs. This is a novel mechanism that underlies the difference between hemorrhagic and non-hemorrhagic SVMPs, improving the understanding of snakebite pathology.

## Author Summary

Snakebite accidents by vipers cause a massive disturbance in hemostasis and tissue damage at the snakebite area. The systemic effects are often prevented by antivenom therapy. However, the local symptoms are not neutralized by antivenoms and are related to the temporary or permanent disability observed in many patients. Although the mechanisms involved in coagulation or necrotic disturbances induced by snake venoms are well known, the disruption of capillary vessels by SVMPs leading to hemorrhage and consequent local tissue damage is not fully understood. In our study, we reveal the mechanisms involved in hemorrhage induced by SVMPs by comparing the action of high and low hemorrhagic toxins isolated from *Bothrops* venoms, in mouse skin. We show remarkable differences in the tissue distribution and hydrolysis of collagen within the hemorrhagic lesions induced by high and low hemorrhagic metalloproteinases. According to our data, tissue accumulation of hemorrhagic toxins near blood vessel walls allowing the hydrolysis of basement membrane components, preferably collagen IV. These observations unveil new mechanistic insights supporting the local administration of metalloproteinases inhibitors as an alternative to improve snakebite treatment besides antivenom therapy.

## Introduction

Snakebite envenoming is an important neglected disease in many tropical and subtropical developing countries. As recently reviewed, globally, venomous snakebite is estimated to affect more than 421,000 humans per year, with 20,000 of fatalities. However, if we take into account the non-reported accidents, these data may be as high as 1,841,000 envenomings and 94,000 deaths [1]. Antivenom therapy was set at the end of 19^th^ century and is still the only efficient approach to treat snakebites. It cures systemic symptoms of envenoming while the local effects are not covered and usually leads to temporary or permanent disability observed in many patients [2], [3]. In Brazil, the majority of the accidents reported to the Ministry of Health are caused by viper snakes [4]. The victims of viper envenoming frequently present systemic disturbances in hemostasis including spontaneous bleeding and blood incoagulability, and strong local effects characterized by edema, ecchymoses, blisters and extensive hemorrhage [2]. Hemorrhagic toxins play an important role in vascular damage and subsequent generation of ischemic areas that largely contribute to the onset of local tissue necrosis that may result in amputation of affected limbs [5], [6].

The pathogenesis of venom-induced hemorrhage involves direct damage of microvessels by the snake venom metalloproteinases (SVMPs). They are multidomain Zn^2+^-dependent proteinases that share structural and functional motifs with other metalloproteinases, such as MMPs (Matrix Metalloproteinases) and ADAMs (A Disintegrin And Metalloproteinase) [7], [8]. SVMPs are classified from PI to PIII according to their domains constitution (Reviewed by Fox and Serrano [9]). The mature form of the PI class is composed only of the metalloproteinase domain with the characteristic zinc-binding site present in all classes of SVMPs, MMPs and some ADAMs. P-II and P-III SVMPs exhibit additional non-catalytic domains, such as disintegrin, disintegrin-like and cysteine-rich domains, similar to those found in ADAMs, which are related to adhesive properties [9]. Despite sharing similar catalytic activity, not all SVMPs induce hemorrhage in *in vivo* models. In general, P-III SVMPs that include disintegrin-like and cysteine-rich domains are potent hemorrhagic toxins while P-I SVMPs show reduced hemorrhagic activity. There are also a number of non-hemorrhagic SVMPs that may be found preferentially in the P-I class and rarely in P-III class, which often function as pro-coagulant enzymes [10], [11], [12].

The mechanism of hemorrhage induced by SVMPs has been investigated in several studies [13], [14], [15], [16], [17]. However, the precise molecular and cellular events associated with microvessel disruption remain unknown. The degradation of vascular basement membrane components has been proposed as a key event for the onset of capillary vessel disruption. The four major components of basement membranes are type IV collagen, laminin, nidogen/entactin and perlecan. Type IV collagen and laminin individually self-assemble into supramolecular structures, and both networks are crucial for basement membrane stability [18]. Nidogen/entactin and perlecan bridge the laminin and type IV collagen networks, increasing their stability, influencing the structural integrity of basement membranes [18], [19]. Thus, the hydrolysis of vascular basement membrane components by SVMPs could profoundly affect the stability of the endothelium, resulting in bleeding. In this regard, *in vitro*, SVMPs efficiently degrade basement membrane components such as laminin, type IV collagen, nidogen/entactin, presenting minor effects on interstitial collagens [20]. However, catalytic activity is apparently similar in hemorrhagic and non-hemorrhagic SVMPs, indicating that the hydrolysis of basement membrane components is not the only mechanism acting on vascular damage induced by the hemorrhagic toxins.

Endothelial cells have also been investigated as potential targets of hemorrhagic toxins. The survival signals promoted by endothelial cell anchorage can be disrupted by SVMPs using mechanisms dependent or independent of their proteolytic activity. Both P-I and P-III SVMPs interfere with adhesion components involved in focal adhesion between endothelial cells and the extracellular matrix, affecting the organization of actin filaments and stress fibers, which culminates in cell death by apoptosis [21], [22]. However, apoptosis of endothelial cells shows little correlation with the hemorrhage induced by SVMPs. Hemorrhagic and non-hemorrhagic SVMPs induce comparable rates of apoptosis in endothelial cells in culture [23] and the onset of hemorrhage induced by SVMPs occurs much earlier than the induction of apoptosis of endothelial cells *in vitro*. In addition, no apoptosis of endothelial cells was observed in SVMP-induced hemorrhage in the dermis of mouse ear skin, *in vivo* [24].

An additional mechanism involved in SVMPs hemorrhagic activity could be related to the accumulation of hemorrhagic SVMPs close to capillary vessels, through the adhesive properties of the non-catalytic domains, allowing the hydrolysis of basement membrane components and disruption of the blood vessels. This mechanism has been suggested previously [14], [16], [25], but up to the moment, there are no experimental evidences that this occurs *in vivo*. In this study, we evaluated this hypothesis analyzing the tissue distribution of jararhagin, a highly hemorrhagic P-III SVMP, and BnP1, a weakly hemorrhagic toxin from P-I class, and the hydrolysis of basement membrane collagen and laminin within the hemorrhagic lesions, using the mouse skin as experimental model. We clearly showed a correlation between the binding of toxins to basement membrane and their ability to induce hemorrhage. Moreover, we showed the *in situ* degradation of basement membrane collagen IV, but not laminin, suggesting that collagen is an important target for the tissue accumulation of hemorrhagic SVMPs.

## Methods

### Ethics statement

The conducts and procedures involving animal experiments were approved by the Butantan Institute Committee for Ethics in Animal Experiments (License number CEUAIB 191/2004).

### Animals

BALB/c mice (18–22 g body weight) were used throughout the study.

### Toxins

As a model for class P-I SVMP with low hemorrhagic activity, we used BnP1 (GI:172044591), a 25 kDa metalloproteinase, isolated from *Bothrops neuwiedi* venom according to Baldo et al., [23], which contains only the catalytic domain. Jararhagin (GI:62468) was used as a model of highly hemorrhagic P-III SVMP, comprised of catalytic, disintegrin-like and cysteine-rich domains. It was isolated from *Bothrops jararaca* venom, as previously described [26]. Jararhagin-C was isolated from *Bothrops jararaca* venom as described [27]. It is devoid of catalytic activity and does not induce hemorrhage, as it contains only the non-catalytic domains of jararhagin – disintegrin-like and cysteine-rich. BSA (Bovine serum albumin) was used as control of non-toxic protein in different set of experiments. Toxins were used in the native form or labeled with Alexa Fluor 488® (Molecular Probes, USA), following the instructions of the manufacturer.

### Histological analysis and detection of extracellular matrix components

Groups of three BALB/c mice were intradermically injected with jararhagin (10 µg) and BnP1 (5 µg or 50 µg), dissolved in 20 µL of PBS (phosphate buffered saline). The control group received only 20 µL of PBS. After 15 minutes, the animals were sacrificed by CO_2_ inhalation and the dorsal skin corresponding to the site of the injection was carefully dissected out and fixed in methacarn solution (60% methanol, 30% chloroform, 10% glacial acetic acid) for 3 hour at 4°C and thereafter dehydrated in ethanol and embedded in Paraplast (Merck, Germany). Sections of 5 µm were adhered to glass slides using 0.1% poly-L-Lysine (Sigma, UK) and dried at room temperature. Sections were dewaxed in xylene and hydrated in distilled water. Each of the succeeding steps was followed by washing with PBS. Some sections were stained with hematoxylin and eosin for histological analysis. The detection of collagen fibers was performed by staining the sections using the picrossirus method, according to a previously described protocol [28].

The staining of basement membrane components was performed by immunofluorescence assays. After dewaxing and hydrating, the slides were submitted to antigen retrieval using enzymatic treatment of the sections with 4 mg/mL solution of pig pepsin (1,120 units/mg protein) (Sigma, UK) in acid buffer (pH 2.2), for 10 minutes at room temperature, followed by incubation with blocking solution (PBS/BSA 10% and goat serum 1∶1) for 1 hour at room temperature. Afterwards, the sections were incubated with goat anti-rabbit type IV collagen polyclonal antibody (Chemicon, USA), at a 1∶40 dilution, or goat anti-rabbit laminin polyclonal antibody (Chemicon, USA), at a 1∶40 dilution, for 18 hours at 4°C. After washing with PBS, the sections were incubated with Alexa fluor 488 goat anti-rabbit IgG (Molecular Probes, USA), at a 1∶1000 dilution, for 90 minutes at room temperature. The nuclear staining was performed with DAPI (4′, 6′-diamino-2-phenylindole, Sigma, UK), at a 1∶1000 dilution. Negative control consisted of omitting the primary antibody step from the protocol. The sections were examined with a Confocal Microscope LSM 510 Meta (Zeiss, Germany).

### Distribution of toxins in skin vasculature

The distribution of toxins in the skin vasculature was evaluated after intradermically injection of 10 µg of Alexa Fluor 488-labeled jararhagin (Alexa488-Jar), 5 µg of Alexa Fluor 488-labeled jararhagin-C (Alexa488-Jar-C) or 50 µg of Alexa Fluor 488-labeled BnP1 (Alexa488-BnP1) in BALB/c mice. The control group received 20 µL of Alexa Fluor 488-labeled bovine serum albumin (Alexa488-BSA). After 15 minutes, the animals were sacrificed by CO_2_ inhalation and the piece of dorsal skin on the site of injection was carefully dissected, frozen in OCT (Optimal cutting temperature) and sectioned 5 µm thick in the cryostat (Leica, CM1510). The sections were then fixed in 3.7% formaldehyde for 10 minutes at room temperature. Nonspecific staining was blocked by incubating the sections for 1 hour at room temperature with PBS containing 1% triton X-100, 5% normal goat serum, 1% BSA, 0.5% glycine and 0.5% fish skin gelatin. Then, the sections were incubated with donkey anti-rat CD-31 polyclonal antibody (BD Bioscience, USA), at a 1∶40 dilution, or goat anti-rabbit type IV collagen polyclonal antibody (Chemicon, USA), at a 1∶40 dilution, for 18 hours at 4°C. The sections were washed with PBS and incubated with TRITC-labeled (Tetramethyl Rhodamine Isothiocyanate) goat anti-rat IgG (Jackson ImmunoResearch, USA), at a 1∶500 dilution, or TRITC goat anti-rabbit IgG (Jackson ImmunoResearch, USA), at a 1∶100 dilution, for 2 hours at room temperature. The distribution of toxins in the tissues was analyzed searching in at least 10 different fields of each section. Alternately, the distribution of jararhagin was analyzed 45 minutes after its injection under the same experimental condition. The sections were examined with a Confocal Microscope LSM 510 Meta (Zeiss, Germany).

## Results

### Morphological alterations induced by SVMPs in mouse skin

In order to investigate the mechanisms involved in the hemorrhage induced by SVMPs, we initially compared the pathological alterations induced by jararhagin, a highly hemorrhagic P-III SVMP and BnP1, a weakly hemorrhagic P-I SVMP, using the mouse skin as experimental model. Tissues were inspected 15 minutes after injection of toxins in order to evaluate the first events involved in the hemorrhagic lesions. At this period, we focused on the direct action of venom toxins, avoiding interference of secondary effects of endogenous components released by the local reaction.

**Figure 1:**
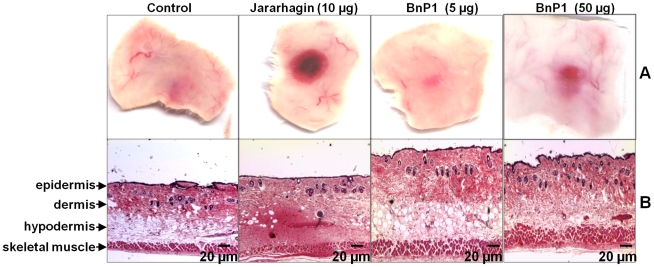
Action of jararhagin and BnP1 in mouse skin. **A-** Macroscopic view of mouse skin 15 minutes after i.d injection of toxins. No hemorrhage was observed in the control samples, whereas the samples injected with jararhagin showed an evident hemorrhagic lesion. BnP1 (5 µg) did not induce bleeding and only a small hemorrhagic lesion was detected when 50 µg of this toxin were used. **B-** Histological analysis of paraffin-embedded sections. Control samples injected only with PBS showed typical histological features of normal skin. The samples injected with jararhagin showed intense hemorrhage especially in the hypodermis region, and was also detected in the skeletal muscle layer. Only an extensive edema was observed in the skin after injection of BnP1 (5 µg). Some hemorrhagic spots were observed after injection of 50 µg of BnP1. Hematoxylin and eosin staining.

After 15 minutes, macroscopic analysis of the skin injected with doses adjusted at the same molar basis (10 µg jararhagin and 5 µg BnP1), revealed that jararhagin induced intense hemorrhage, whereas only a small hemorrhagic spot at the site of the injection was observed in the samples injected with BnP1 or PBS, used as injection control (Fig. 1A). Only high doses of BnP1 (50 µg) was able to induce hemorrhage, but less intense than jararhagin (Fig. 1A). Morphological analysis under light microscopy showed drastic hemorrhage in the hypodermis and also in the skeletal muscle adjacent to hypodermis in mice injected with jararhagin (Fig. 1B). The equivalent dose of BnP1 induced an enlargement of skin thickness probably due to its edema-forming activity but did not induce hemorrhagic alterations. When a hemorrhagic doses of BnP1 (50 µg) was injected, edema was persistent and only sparse spots of hemorrhage were detected in the hypodermis (Fig. 1B). We also evaluated the action of jararhagin and BnP1 on dermal-epidermal junctions by staining with antibodies anti-laminin and anti-β_4_ integrin and no alteration of these structures were observed after injection of toxins (data not shown). These results confirm the differences in hemorrhagic activity of jararhagin and BnP1 and show that most of the hemorrhagic incidence occurs in the hypodermis.

### *In vivo* degradation of extracellular matrix components induced by SVMPs

**Figure 2:**
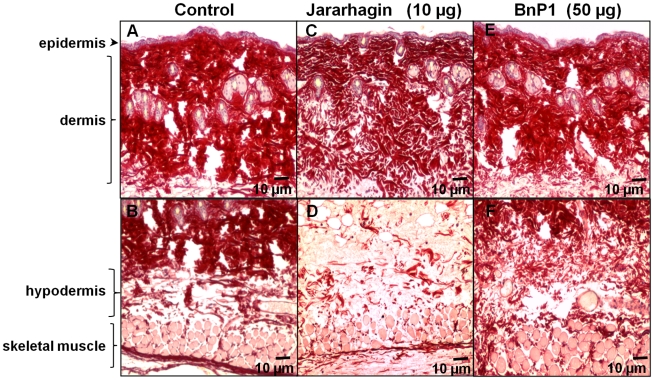
Action of jararhagin and BnP1 on the fibrillar collagen matrices. Paraffin-embedded sections of mouse skin 15 minutes after i.d injection of 10 µg jararhagin and 50 µg of BnP1. Control samples injected with PBS showing the usual distribution of collagen forming closely packed bundles of fibers (A, B). Jararhagin injected samples showed a clear loosening of the bundles of collagen fibers in the dermis (C) and fragmented collagen fibers in the hypodermis region (D). Samples injected with BnP1 showed a moderate disorganization of collagen fibers throughout the dermis (E, F). Picrossirius method staining.

Next, we investigated the integrity of extracellular matrix components, mainly collagens and laminin, after injection of hemorrhagic doses of jararhagin (10 µg) or BnP1 (50 µg). After 15 minutes, the control skin injected with PBS, showed a dense network of collagen fibers stained by picrossirus. The bundles of collagen fibers were closely packed characterizing a dense connective tissue (Fig. 2 A, B). In contrast, mice injected with jararhagin showed a clear loosening of the bundles of collagen fibers in the dermis (Fig. 2C). In the hypodermis, where the hemorrhagic lesion occurs, only a few weakly stained fibers were observed, indicating a massive degradation of fibrillar collagen (Fig. 2D). BnP1 induced only a discrete disorganization of collagen fibers throughout the dermis (Fig. 2E) and the hypodermis (Fig. 2F).

**Figure 3:**
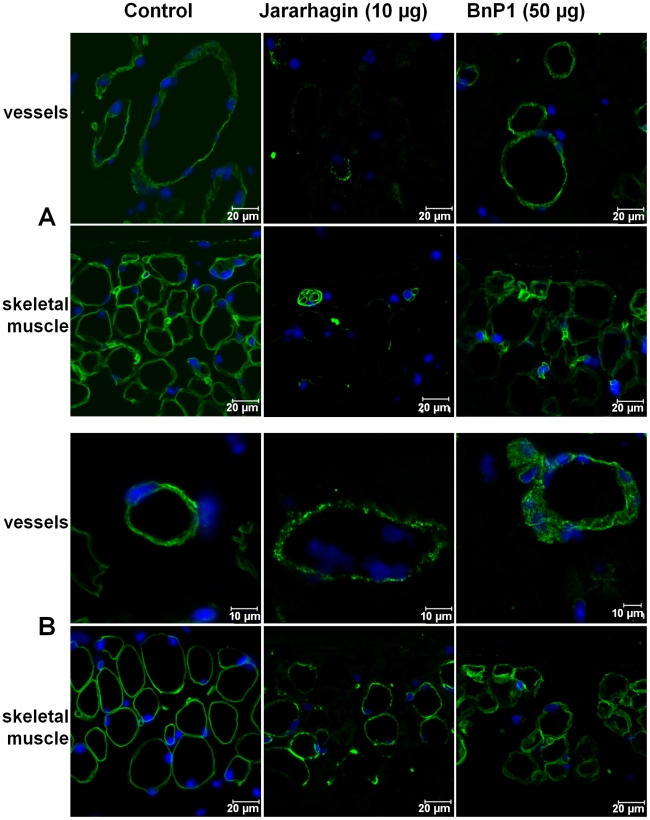
Action of jararhagin and BnP1 on basement membrane components. Paraffin-embedded sections of mouse skin 15 minutes after i.d injection of 10 µg of jararhagin and 50 µg of BnP1. Control samples were injected with PBS. **A-** Immunofluorescence for type IV collagen. Control samples showed a continue immunoflurescent line surrounding small venules and skeletal muscle cells (green). Jararhagin induced remarkable degradation in the immunostaining of type IV collagen in the basement membrane of blood vessels and skeletal muscle. BnP1 induced only a moderate disorganization of type IV collagen in the basement membrane. **B-** Immunofluorescence for laminin. Control samples showed the usual distribution of laminin in the basement membrane (green). Jararhagin and BnP1 induced slight alteration in the distribution of laminin in the basement membrane of blood vessels and skeletal muscle cells. The nuclear staining was performed with DAPI (blue). The sections were examined with a Confocal Microscope LSM 510 Meta (Zeiss).

The effect of toxins on the distribution of type IV collagen and laminin at the basement membrane was then evaluated by immunofluorescence. In the control skin, type IV collagen (Fig. 3A) and laminin (Fig. 3B) were observed as linear and continuous lines surrounding small blood vessels and in the basement membrane of skeletal muscle cells. In contrast, jararhagin induced a remarkable alteration in the immunostaining of type IV collagen in the basement membrane of blood vessels of the hypodermis, where only traces of type IV collagen deposition were detected. In addition, jararhagin also promoted a notable reduction of type IV collagen immunostaining in skeletal muscle basement membrane (Fig. 3A). After injection of 50 µg of BnP1, only a slight alteration in the immunostaining of type IV collagen was observed. The immunoreaction in the basement membrane of blood vessels and skeletal muscle was more diffuse than the pattern observed in the control, with the presence of some spots. However, the alterations induced by BnP1 are not comparable to the extensive disruption induced by jararhagin on type IV collagen, whose immunoreaction was practically abolished in blood vessels and skeletal muscle basement membrane (Fig. 3A). The effect of jararhagin on laminin distribution was less intense than that observed on type IV collagen. The presence of laminin was detected in tissues injected with jararhagin, but its distribution on basement membrane was not as homogeneous as observed in control tissues suggesting punctual disruptions of basement membrane integrity, probably as a result of collagen degradation. Similar effects were induced by BnP1 (Fig. 3B).

### Distribution of toxins in the skin vasculature

**Figure 4:**
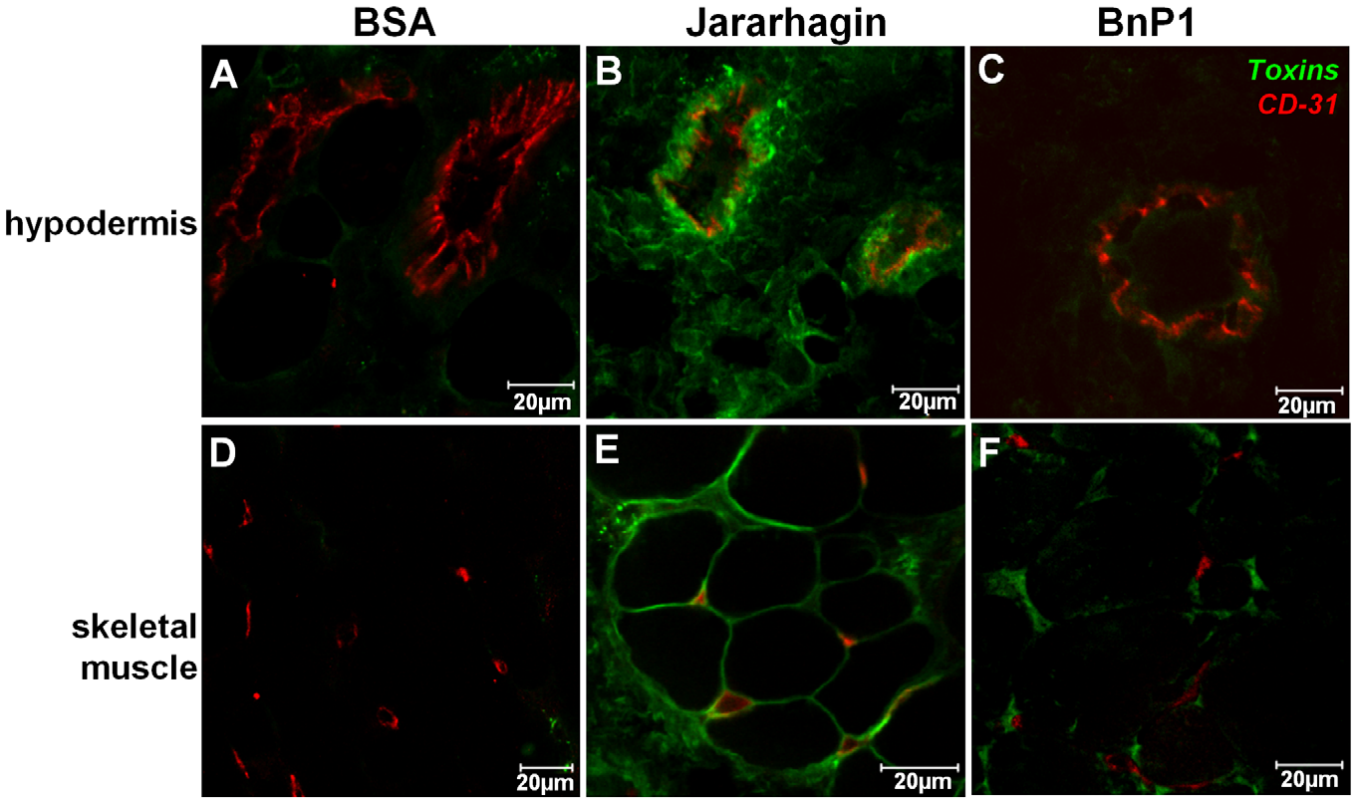
Distribution of Alexa488-labeled SVMPs on the skin vasculature. The cryosections were obtained 15 minutes after i. d. injection of 10 µg of Alexa488-Jar and 50 µg Alexa488-BnP1 in mouse skin. Samples injected with Alexa488-BSA were used as a control. The blood vessels were stained with anti-rat CD-31 polyclonal antibody (red). Alexa488-Jar accumulated in the venules walls in the hypodermis region (B-green) as well as on the basement membrane of skeletal muscle cells and capillaries (E). Alexa488-BSA (A, D) and Alexa488-BnP1 (C, F) did not concentrate around these structures. The sections were examined with a Confocal Microscope LSM 510 Meta (Zeiss).

**Figure 5:**
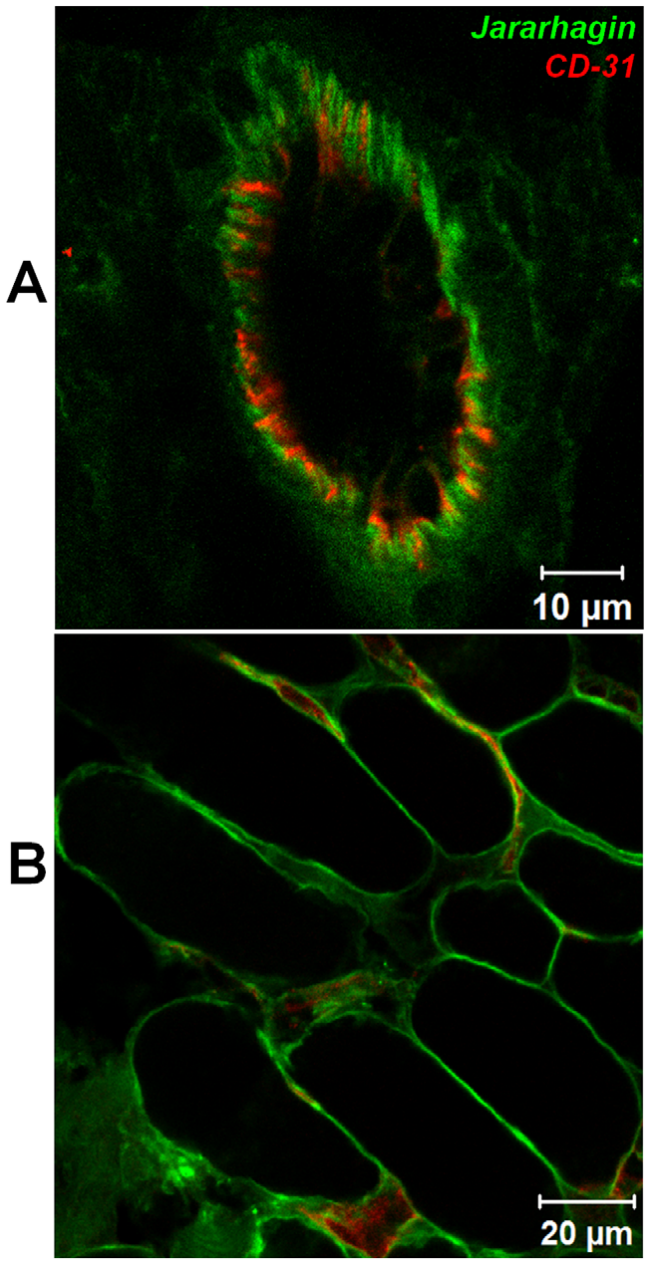
Distribution of Alexa488-Jar on the skin vasculature. Cryosections were obtained 45 minutes after injection of 10 µg of Alexa488-Jar in mouse skin. Alexa488-Jar accumulated in the wall of venules (A-green) and capillaries (B), which were stained with CD-31 blood vessel marker (red). The sections were examined with a Confocal Microscope LSM 510 Meta (Zeiss).

The next step was to analyze the distribution of SVMPs in the skin tissue. After 15 minutes of injection, when the hemorrhagic lesion has already been set, jararhagin was located close to small blood vessels stained by anti-CD31, in the hypodermis region (Fig. 4B). It is interesting to note that co-localization with CD-31 was not observed, suggesting the accumulation of the toxin near the blood vessels. In contrast, after injection of BnP1 (Fig. 4C), only a weak and diffuse fluorescence was observed, slightly higher than in control tissues (Fig. 4A). Similar deposition of the toxins was observed in the skeletal muscle adjacent to the hypodermis. High fluorescence was observed after jararhagin injection (Fig. 4E) in the basement membrane of skeletal muscle and capillaries suggesting its accumulation in these areas. After BnP1 injection (Fig. 4F), only a weak fluorescence was observed. No fluorescence was detected in control tissues (Fig. 4D). Similar pattern of toxin distribution was detected up to 45 minutes after jararhagin injection in areas adjacent to the main focus of the injection. This toxin was detected around hypodermis blood vessels (Fig. 5A), and close to the capillaries in the skeletal muscle (Fig. 5B).

**Figure 6:**
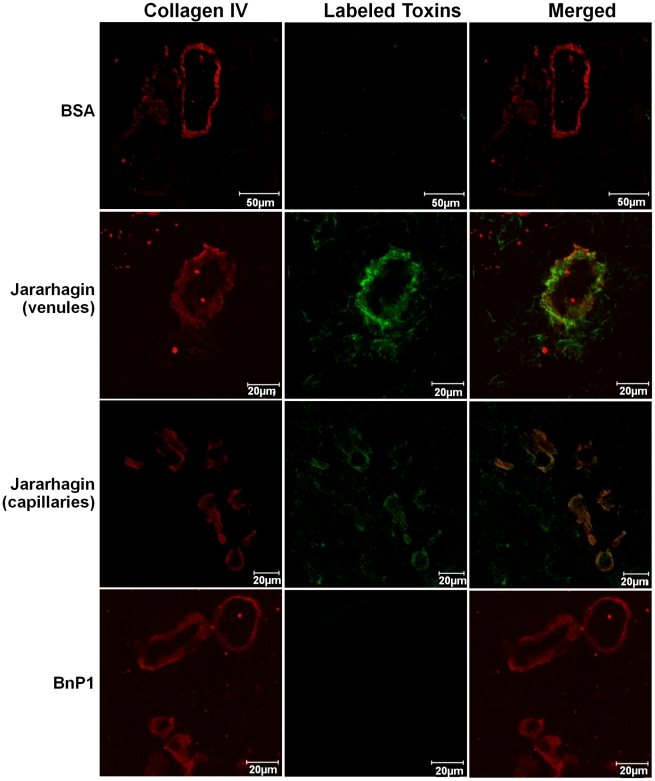
Co-localization of Alexa88-labeled toxins with basement membrane. Cryosections were obtained 15 minutes after injection of 10 µg of Alexa488-Jar and 50 µg of Alexa488-BnP1 in mouse skin. Alexa488-Jar showed co-localization with the basement membrane staining with type IV collagen (red) in the venules and capillaries walls (green). Alexa488-BnP1 did not co-localize with type IV collagen neither in venules nor in capillaries showing similar pattern observed to Alexa488-BSA (control). The sections were examined with a Confocal Microscope LSM 510 Meta (Zeiss).

In order to verify the binding of hemorrhagic toxins to the basement membrane, we carried out a double-staining protocol using Alexa488-labeled SVMPs and type IV collagen antibody. According to figure 6, jararhagin showed co-localization with type IV collagen in the basement membrane of venules and capillaries, 15 minutes after injection. Contrarily, no co-localization with type IV collagen was observed in mouse skin injected with BnP1, which showed a similar staining pattern to control samples (Fig. 6). These results confirm the particular binding of hemorrhagic toxins to basement membrane components, explaining their accumulation near blood vessels.

**Figure 7:**
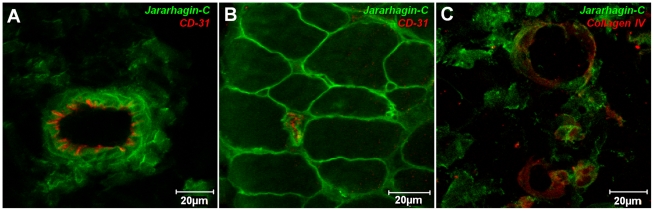
Role of disintegrin-like and cysteine-rich domains on the distribution of jararhagin on skin vasculature. Cryosections were obtained 15 minutes after injection of 5 µg Alexa488-Jar-C in mouse skin. Alexa488-Jar-C accumulated on venules (A) and capillaries walls (B), and showed co-localization with basement membrane staining with type IV collagen (C-red). The sections were examined with a Confocal Microscope LSM 510 Meta (Zeiss).

We next addressed the role of non-catalytic domains of SVMPs in the distribution of jararhagin on tissues. For that, mice were injected with Alexa488- labeled jararhagin-C, which consists of jararhagin disintegrin-like and cystein-rich domains, and its distribution was observed in mouse skin. The distribution of jararhagin-C in skin was the same for jararhagin: jararhagin-C was detected close to the CD-31 marker around blood vessels in the hypodermis (Fig. 7A), and close to capillaries in the skeletal muscle (Fig. 7B), and co-localized with basement membrane type IV collagen in the hypodermis venules (Fig. 7C). These results strongly suggest that the non-catalytic domains are determinant to hemorrhagic activity of SVMPs from P-III class, locating the catalytic site specifically to the microvascular wall.

## Discussion

In this work, we unveil an important step for understanding the mechanisms involved in the expressive hemorrhage induced by SVMPs, by comparing the *in vivo* degradation of extracellular matrix proteins and the tissue distribution of jararhagin, a highly hemorrhagic P-III SVMP, and BnP1, a weakly hemorrhagic P-I SVMP, using the mouse skin as experimental model. This comparison revealed that tissue localization and *in vivo* degradation of collagens are key events in SVMPs induced hemorrhage.

Jararhagin induced a massive degradation of fibrillar collagen in the hypodermis, where the hemorrhagic lesion was concentrated. In the vascular basement membrane, type IV collagen was the major substrate for jararhagin. In contrast, BnP1 was not able to efficiently degrade these substrates or to induce hemorrhage. Instead, a remarkable edema and dermal alteration were observed, consistent with the dermonecrotic activity already described for BaP1, a SVMP class P-I isolated from *B. asper* venom [29], . Considering that the macromolecular organization and the biomechanical stability of basement membrane are mainly determined by the type IV collagen network [31], its cleavage would alter the structural stabilization of the other related basement membrane components. Consistent with that, we observed that jararhagin induced only slight alterations in laminin distribution, suggesting that its epitopes are conserved after treatment with the toxin**.** This characteristic is not restricted to snake venom pathology. Selective degradation of type IV collagen has been associated with other pathologies involving toxic metalloproteinases. The metalloproteinase from *Vibrio vulnificus* (VVP) is a major determinant for skin lesions of this microorganism, which also causes hypodermic hemorrhage [32].

According to the literature, both P-I and P-III SVMPs are similarly able to hydrolyze extracellular matrix components *in vitro*, such as matrigel [33] and isolated components, such as type IV collagen, laminin and fibronectin [20], [33]–[37]. Escalante et al. [17] showed that jararhagin and BaP1 had a similar proteolytic activity on matrigel with a slightly different cleavage pattern, since BaP1, exerted a limited proteolysis of both laminin and nidogen, whereas jararhagin predominantly degraded nidogen. However, the hydrolysis of extracellular matrix components *in vitro* occurs only after long incubation periods, suggesting that distinct mechanisms are involved in the basement membrane digestion *in vivo*. These authors also analyzed the immunostaining of laminin, nidogen, type IV collagen and the endothelial cell marker VEGFR-2 (vascular endothelial cell growth factor receptor 2) in mouse gastrocnemius muscle injected with hemorrhagic doses of jararhagin and BaP1, observing reduction in the number of capillary vessels and a similar pattern of immunostaining for the basement membrane components laminin, nidogen and type IV collagen in muscular fibers after injection of BaP1 or jararhagin, showing a disorganization of extracellular matrix [17]. Although they showed the first evidence of the catalytic action of SVMPs *in vivo* and morphological alterations in muscular tissues, the authors failed to detect any difference between weakly and highly hemorrhagic SVMPs.

A parameter not yet explored in the literature consisted of eventual differences in the distribution of toxins in the damaged tissues. In this study, jararhagin, but not BnP1, concentrated in the vicinity of small venules and in capillaries of skeletal muscle. This effect was correlated to the non-catalytic domains of jararhagin. *In vitro*, SVMPs bind to extracellular matrix proteins, such as type I collagen [15], [38], [39], [40], type IV collagen [40], collagen XII and XIV and the matrilins 1, 3 and 4 [16]. The high affinity for these extracellular matrix proteins could contribute for the accumulation of the toxin in the damaged tissue, enhancing the catalytic action of SVMPs towards basement membrane components.

A fine correlation between collagen binding and hemorrhagic activity has been shown by our group. An anti-SVMP monoclonal antibody neutralizes hemorrhagic activity and collagen binding of jararhagin without interfering with its catalytic activity [25]. Recently, it was shown that jararhagin binds with high affinity to type I collagen and type IV collagen, whereas berythrativase, a non-hemorrhagic P-III SVMP isolated from *B. erythromelas* venom, failed to bind to these substrates [40]. Molecular modeling of the putative epitopes binding to this monoclonal antibody pinpointed a motif present in the hemorrhagic toxin jararhagin and absent in the pro-coagulant enzyme berythractivase, located at the Da-subdomain of disintegrin-like domain [40]. However, it is important to consider that a collagen-binding motif was also detected in the cysteine-rich domain of Atrolysin-A, a hemorrhagic P-III SVMP [15].

One aspect still unclear is the apparent contradiction between the results of collagen hydrolysis *in vitro* and in *vivo*. *In vitro*, most SVMPs (class P-I or P-III) hydrolyse type IV collagen but not fibrillar collagens [20]. Here, an almost complete disassembly of hypodermal fibrillar collagen was observed in tissues treated with jararhagin, but not BnP1. Escalante and co-workers [41] also observed collagen hydrolysis *in vivo.* Several fragments of collagens were detected in the exudates of muscle injected with BaP1, a class P-I SVMP. Most of the fragments corresponded to non-fibrillar collagen. However, a fragment corresponding to the fibrillar collagen V was also identified. According to our results, class P-I SVMPs induced minor alterations to hypodermal fibrillar collagen. Thus, it is possible to predict that skin homogenates of P-III SVMP lesions would contain more degradation fragments of fibrillar collagens. A possible explanation for the differences observed *in vivo* could be the disorganization of fibrils due to the toxin binding allowing the degradation of fibrillar collagen on hypodermis. However, data explaining why this occurs only *in vivo* is still lacking. Other alternative would be that the injection of SVMPs would induce tissue secretion of MMPs, able to digest fibrillar collagen. However, this hypothesis is not consistent with the fast onset of the reaction. Also, P-I SVMPs are very efficient to induce over-expression of MMPs [29], [42] and they appear to be much less efficient to hydrolyze collagen *in vivo*.

Our results confirm previous suppositions that the non-catalytic domains play a crucial role in the expression of hemorrhagic activity of P-III SVMPs, implying that non-enzymatic mechanisms are also involved in bleeding. The hypervariable region of the cysteine-rich domain is attributed to the binding of SVMPs to a series of substrates containing von Willebrand factor A domains, allowing the catalysis of a specific substrate region [16]. Here we suggest that SVMPs may present additional functional motifs related to their binding to collagens. The cysteine-rich domain exosite would be essential for the enzymatic selectivity of the SVMPs while a disintegrin-like domain collagen binding motif would be responsible for high affinity binding to collagens and tissue concentration of the toxin.

The data presented herein are particularly important to understand the mechanisms involved in the onset of hemorrhage and could contribute to the rational of alternative treatments for snakebites victims. Since accumulation of SVMPs at the site of the bite allows hemorrhage enhancing the local venom effects, the local administration of metalloproteinases inhibitors could represent an interesting approach in order to improve the neutralization of toxins responsible for the local damage. Indeed, it has already been shown that the local injection of batimastat, a peptidomimetic matrix metalloproteinase inhibitor, totally neutralized the proteolytic, hemorrhagic and dermonecrotic effects induced by *Bothrops asper* venom [43]. Moreover, it has recently been shown that local administration of tetracycline prevented the dermonecrosis induced by *Loxosceles* spider venom. In addition to its antimicrobial properties, tetracycline was also able to inhibit MMPs, which are important for the progression of dermonecrotic lesions [44].

In summary, we showed that the strong hemorrhage induced by class P-III hemorrhagic SVMPs is related to their accumulation at basement membrane, reaching enzyme concentrations sufficient for its rapid degradation. This mechanism may serve as a rational for the design of alternatives in which local administration of metalloproteinase inhibitors may complement antivenoms in the neutralization of local tissue damage.
